# The Relationship Between Healthy Eating Motivation and Protein Intake in Community-Dwelling Older Adults With Varying Functional Status

**DOI:** 10.3390/nu12030662

**Published:** 2020-02-28

**Authors:** Hanna M. Rempe, Gudrun Sproesser, Anne Hannink, Thomas Skurk, Beate Brandl, Hans Hauner, Britta Renner, Dorothee Volkert, Cornel C. Sieber, Ellen Freiberger, Eva Kiesswetter

**Affiliations:** 1 Institute for Biomedicine of Aging, Friedrich-Alexander-University of Erlangen-Nürnberg, Kobergerstraße 60, 90408 Nürnberg, Germany; Anne.Gingrich@fau.de (A.H.); Dorothee.Volkert@fau.de (D.V.); Cornel.Sieber@fau.de (C.C.S.); Ellen.Freiberger@fau.de (E.F.); Eva.Kiesswetter@fau.de (E.K.); 2 Psychological Assessment and Health Psychology, University of Konstanz, Box 47, 78457 Konstanz, Germany; gudrun.sproesser@uni-konstanz.de (G.S.); britta.renner@uni-konstanz.de (B.R.); 3 Else-Kröner-Fresenius-Center for Nutritional Medicine, Technical University of Munich, Gregor-Mendel-Straße, 85354 Freising-Weihenstephan, Germany; skurk@tum.de (T.S.); hans.hauner@tum.de (H.H.); 4 ZIEL Institute for Food and Health, Core Facility Human Studies, Technical University of Munich, Gregor-Mendel-Str. 2, 85354 Freising, Germany; beate.brandl@tum.de; 5 Klinikum Rechts der Isar, Institute of Nutritional Medicine, Technical University of Munich, Georg-Brauchle-Ring 62, 80992 Munich, Germany; 6 Department of Medicine, Kantonsspital Winterthur, Brauerstraße 15, 8400 Winterthur, Switzerland

**Keywords:** protein intake, protein sources, healthy eating motivation, older adults, functional status

## Abstract

In older adults, the relationship between healthy eating motivation (HEM) and protein intake as key component of a healthy diet is poorly understood. Therefore, we investigate the association of HEM with (1) total protein intake and (2) intake of different protein sources in older adults with varying functional status. In this cross-sectional study including 250 adults (≥70 years), we assessed HEM with “The Eating Motivation Survey” and protein intake by 7-day food records. In addition, gender, age, Body Mass Index (BMI), energy intake and functional status were considered. Regression analyses revealed that HEM was neither related to total (*β* = −0.02; *p* = 0.723) nor to relative protein intake (*β* = 0.04; *p* = 0.370). Notwithstanding this, participants with stronger HEM showed lower intake of protein from meat and meat products (*β* = −0.14; *p* = 0.018), higher intake of overall plant-based protein (*β* = 0.11; *p* = 0.032), protein from fruit and vegetables (*β* = 0.20; *p* = 0.002) and from pulses, nuts an seeds (*β* = 0.16; *p* = 0.016). Our findings suggest HEM as a valuable indicator for intake of distinct protein sources. However, since HEM is not related to total protein intake, the importance of sufficient protein consumption should be emphasized by promoting healthy eating, regardless of self-perceived HEM.

## 1. Introduction

A balanced diet as part of a healthy lifestyle supports healthy aging and might counteract the increased risk of declining physical function and mobility with age [1]. Effective modification of dietary behavior requires knowledge about the link between underlying motives and eating patterns. Of special interest is the association between health concerns in terms of diet and actual eating behavior, as it seems intuitive that stronger healthy eating motivation (HEM) leads to choosing health-promoting foods. This assumption is supported by findings from previous studies that have identified HEM as a factor for higher intake of fruits and vegetables and lower consumption of energy-dense foods [2,3,4,5].

Although a healthy diet at all ages consists of the interplay between need-based energy and macro- and micronutrient intake, protein intake in older adults has gained specific attention because of its role in muscle metabolism [6]. Due to inflammation, insulin resistance, and reduced postprandial availability of amino acids, it appears that the muscles of older persons are less responsive to the anabolic stimulus of dietary protein for muscle protein synthesis (MPS). Consequently, a higher amount of protein may be required to optimally stimulate postprandial MPS compared to the young [7]. A number of studies suggest that protein intake of at least 1.0 g per kg body weight [g/kg BW] is necessary to maintain muscle health and to prevent functional decline, sarcopenia, and disability [8,9,10]. Nevertheless, insufficient protein intake is still prevalent, especially in the “oldest–old” and older adults with functional limitations [11,12,13]. A body of literature highlights the interaction between functional status and protein consumption by identifying a poor functional status as both a consequence and a risk factor of low protein intake [10,14,15]. In addition to the amount of consumed protein, the quality of protein seems to be a factor for maintaining muscle health [7,16]. Findings from previous studies showed that animal-based protein (e.g., dairy or meat-based protein) is more potent in stimulating MPS compared to plant-based protein [17], due to the composition of essential amino acids, the amount of leucine, and the digestibility.

Although prior studies concordantly identified a generally high priority of health concerns in food choice among older persons [18,19], to the best of the authors’ knowledge, no study has examined the association of HEM with intake of total protein and different protein sources. HEM could be both a driver and barrier for protein consumption, as people may have a different understanding of which protein-foods are part of a healthy diet. Since a strong HEM promotes consuming plant-based, unprocessed foods [20], there might also be a connection with comparatively low animal protein intake [5] and lower total protein consumption. Mendonça et al. [12] showed that a higher contribution of meat and meat products to total protein intake was associated with a reduced likelihood of low protein intake in community-dwelling older people. In addition, misconception and misinformation about potentially harmful effects of certain protein-rich foods could lead to their avoidance in health-conscious older adults. For example, a reduced consumption of milk and dairy products was recommended in terms of reducing the cardiometabolic risk, although current evidence from epidemiologic and intervention studies does not entirely support this [21,22,23]. However, as the above described health-benefits of a protein-rich diet become increasingly part of health promotion campaigns for older people, a positive relationship between HEM and total protein intake is also conceivable.

Gaining a better understanding of the association of health concerns in food choice with protein consumption could serve as a basis to modify protein intake. Therefore, the present study aims at investigating the association of HEM with (1) total protein intake and (2) intake of different protein sources among older adults with varying functional status.

## 2. Materials and Methods

### 2.1. Study Design and Participants

This analysis is based on two cross-sectional studies (SPRINTT-trial and *enable*-study) in community-dwelling, older adults aged ≥70 years with different functional status. Recruitment took place between February 2016 and December 2017 via advertisements in the local media (e.g., radio, newspaper) and by using addresses and phone numbers of the citizens registry of the cities of Nuremberg and Freising, Germany. In both studies, a Mini Mental Status Examination (MMSE, 0–30) score ≥24 served as an inclusion criterion to select older people without cognitive impairment [24]. Based on further study, specific in- and exclusion criteria of participants of the studies differed with regard to physical functional status.

The SPRINTT sample consisted of 124 persons who showed poor or fair functional status after a first screening visit at the study center of the University of Erlangen-Nürnberg. Further inclusion criteria were self-reported difficulties in daily activities (e.g., walking, climbing stairs) and the presence of low muscle mass. Illnesses like cancer, severe arthritis, lung disease, or progressive neurological disorder led to exclusion. Detailed descriptions of inclusion criteria and study design of the European SPRINTT-trial are provided elsewhere [18,25].

The *enable* sample comprised 149 healthy older persons who were recruited in regions of Nuremberg and Freising. Exclusion criteria to generate a healthy sample were the presence of acute/chronic disease, a BMI less than 18.5 or greater than 35 kg/m^2^, smoking, immobility, need of care, unintended weight loss of more than 5% in the previous three months, and blood transfusion in the previous three months. For further information on the exclusion criteria of the *enable*-study, see Gingrich et al. [26,27].

The *enable*-sample only included participants within the age-range 75–85 years, with nearly equal proportions of women and men, whereas the SPRINTT-trial included participants from the age of 70 years and had no restrictions regarding maximum age or proportions of women and men. For the present analysis, only participants with complete dietary data were included.

### 2.2. Ethics, Consent and Permissions

The studies were performed according to the guidelines published in the Declaration of Helsinki and the study protocols were approved by the ethics committees of the Friedrich-Alexander-Universität Erlangen-Nürnberg and of the medical faculty of the Technical University of Munich, Germany, respectively. Written informed consent was obtained from every participant prior to the start of the assessments. Both studies were registered (SPRINTT-trial: ClinicalTrials.gov identifier: NCT02582138; *enable*-cluster: German Clinical Trials Register DRKS-ID: DRKS00009797).

### 2.3. Measures

#### 2.3.1. Sociodemographics and Anthropometry

Gender, age, and living-status (living alone, yes vs. no) were assessed through a standardized questionnaire. Body weight in light clothing and height were measured, referring to standard operating procedures and used to calculate BMI (kg/m^2^).

#### 2.3.2. Healthy Eating Motivation (HEM)

HEM was assessed by the eating motive “Health” subscale of the brief Eating Motivation Survey (TEMS, [28]), which has already shown to be invariant among older persons varying in functional status [18,28]. The brief health scale has been shown to be reliable (Cronbach’s alpha = 0.81; [28]) and valid, displaying high convergent validity with the health motive from the Food Choice Questionnaire [29], with *r* = 0.57, *p* < 0.001 [30].

The TEMS health motive is represented by three single items (“I eat what I eat … (1)…to maintain a balanced diet; (2) …because it is healthy; (3) …because it keeps me in shape, e.g., energetic, motivated”), each rated on a scale from 1 “never” to 7 “always”. A combined mean score of the three items was calculated, which represents HEM within a range of 1 “eating is never health-motived” to of 7 “eating is always health-motivated”.

#### 2.3.3. Functional Status

Functional status was evaluated with the short physical performance battery (SPPB) testing three different domains of physical function: balance (side-by-side, semi-tandem, tandem stand), usual gait speed (4 m), and strength in lower extremities (5-repetition sit-to-stand) [31]. As suggested by Guralnik et al. [32], a sum score (0–12) was calculated with a higher overall sum score indicating better physical performance. The study sample represents a great heterogeneity in terms of functional status by including participants with an SPPB sum score ranging from 3–9 in the SPRINTT-trail and 10–12 in the *enable*-study. For the analyses in this paper, a continuous SPPB sum score was used.

#### 2.3.4. Protein Intake

Current dietary intake was assessed using an estimated consecutive 7-day-food record. The food records did not include predefined food groups but had open fields to list all foods eaten. Trained study personnel instructed the participants both verbally and in written form and was available to answer questions. Participants were advised to stick to their usual eating habits and to report all consumed food items and beverages as detailed as possible, including portion sizes (grams, usual household measures, packaging information), fat content (e.g., milk, yoghurt), cooking methods, and time of consumption. In case the participants prepared their meals according to recipes, they were instructed to report the individual ingredients and quantities. To avoid an effect of the season on dietary intake, recruitment was spread over the different seasons. In addition, food records were not collected over public holidays such as Christmas, which might strongly influence eating behavior.

All 7-day food records were checked by a nutrition scientist or a dietitian for completeness and plausibility. If required, the participants were contacted and missing information was requested. Mean values of daily intakes of energy (kilocalories (kcal)), fat and carbohydrates (gram (g)), as well as protein (gram (g), gram per kilogram body weight (g/kg BW)) were calculated for the recorded 7 days using the EBISpro-software (EBISpro, Willstätt-Legelshurst, Germany, 2016), based on the German nutrient database “Bundeslebensmittelschlüssel” (Version 3.02, Karlsruhe, Germany). All entered data were cross-checked by another nutrition scientist.

To investigate protein intake by different food sources, we assigned the calculated protein content of each reported food item to one of eight protein sources, respectively. Our approach was based on the procedure of a prior study, according to Gingrich et al. [26], but due to the small proportions to total protein intake, we combined some of the protein sources, resulting in 8 categories. Correspondingly, four animal protein sources (“meat and meat products”, “dairy products”, “fish and seafood”, “other animal-based protein sources” (combining the protein sources “eggs and egg products”, “pastry, confectionery, predominantly animal-based”, and “other predominantly animal-based protein sources”)) and four plant protein sources (“starchy foods”, “fruits and vegetables”, “pulses and nuts and seeds”, and “other plant-based protein sources” (combining the protein sources “pastry, confectionery, predominantly plant-based”, and “other predominantly plant-based protein sources”) were considered.

### 2.4. Statistical Analyses

Statistical analysis was performed by using SPSS Version 25 (IBM SPSS Statistics, Chigaco, IL, USA).

Participants’ characteristics are presented as mean ± standard deviation (SD) for continuous variables. Dichotomous and categorical variables are shown as absolute numbers and percentages. Chi-square tests or independent *t*-tests were applied to test for significant differences between women and men. Furthermore, we investigated associations between dietary intake variables by using Pearson correlation analyses.

Prior to the regression analyses, data were checked for normality (Q-Q-Plots, histograms) and outliers (standardized residuals, Cook’s distance). Multiple hierarchical linear regression analyses were performed to investigate the association of HEM and variables representing intake of total protein (g), relative protein (g/kg BW), animal protein (g), plant protein (g), and protein by eight subgroups of animal or plant sources (g), respectively.

In each of the 12 hierarchical regressions, HEM was the first variable entered in Model 1, followed by functional status in Model 2. Since, to our knowledge, this is the first study to investigate the relationship between HEM and protein intake in older adults with varying functional status, the interaction term between HEM and functional status was included in Model 3. However, the interaction term was only retained in the respective model if it showed a significant association with the respective dependent variable. Model 4 was adjusted for gender (dummy coded, 0 = female, 1 = male), age, BMI, and energy intake. Pearson correlations coefficients (*r*) of all independent variables (see Supplementary Materials, Table S1) and a variance inflation factor (VIF) of 1.24 indicated that a low level of multicollinearity was present. Follow-up analyses testing for significant simple slopes in interactions and calculating Johnson–Neyman significance regions were conducted with the SPSS-macro “PROCESS 4.3” [33].

Results for chi-square tests or independent *t*-tests were considered statistically significant at a Bonferroni-adjusted *p*-value of <0.002. The level of significance for regression analysis was set at *p* < 0.05 and additionally evaluated based on 95% confidence intervals (CI). 

## 3. Results

### 3.1. Participants’ Characteristics

Of 273 eligible individuals, 22 SPRINTT-participants were excluded due to missing data on dietary intake. In addition, one male participant of the enable-study was excluded because of a mean daily energy intake (416.7 kcal/day) of more than 3 SD below the mean of the male participants. In total, a group of 250 participants was analyzed. Compared to the participants of the complete-cases sample, the participants of the drop-out sample showed significantly worse functional status according to the SPPB sum score (*p* < 0.001). For all other descriptive variables of interest, no significant differences emerged (see Supplementary Materials, Table S2).

Participants’ characteristics are presented in Table 1 for the total sample and separately for women and men. Mean age of the participants was 79.3 ± 4.2 years. More than half of the participants lived alone, with a significantly higher proportion among women (*p* = < 0.001). The SPPB sum score of 9.6 ± 2.5 indicated, on average, a fair functional status. Mean daily energy intake was 1806.1 ± 418 kcal with an expected significantly higher intake of male participants (*p* = < 0.001, see Table 1). In line with this, intake of the analyzed macronutrients carbohydrates, fat, and protein was higher in men (*p* = < 0.001).

### 3.2. Descriptive Results of Protein Intake and HEM

Participants showed a mean total protein intake of 67.6 ± 17.7g and a relative protein intake of 0.9 ± 0.3 g/kg BW, respectively (see Table 1). Relative protein intake was below 0.8 g/kg BW in 30.4% of participants. As presented in Table 1, men consumed significantly more animal-based protein, particularly from the source “meat and meat products” (*p* < 0.001), and protein from other plant-based protein sources compared to women (*p* < 0.001). Pearson correlation analyses of dietary intake variables showed no significant association between intake of plant-based protein and animal-based protein (see Table 2). As shown in Table 2, significant correlations within the eight distinct protein sources ranged from −0.19 (“other animal-based protein sources” and “fruits and vegetables”, *p* < 0.01) to 0.25 (“fruits and vegetables” and “pulses and nuts and seeds”), indicating low to moderate associations.

Mean score of HEM was 4.9 ± 1.5 without significant gender differences.

### 3.3. Relationship Between HEM and Total Protein Intake

Results for adjusted Model 4 are displayed in Table 3 and Table 4, respectively. Univariate results, the single steps of hierarchical regression analysis, and *R*^2^-changes are presented in Table S3 in the Supplementary Materials.

HEM did not show a significant association with daily total protein intake (g) and relative protein intake (g/kg BW), taking functional status and possible confounders, namely, age, gender, BMI and energy intake, into account (see Table 2). Functional status was significantly related to relative protein intake (*β* = −0.11, *p* = 0.023), with functionally fitter participants having a lower mean intake.

### 3.4. Relationship Between HEM and Protein Sources

Regarding the protein sources, significant main effects of HEM on overall plant-based protein intake (*β* = 0.11, *p* = 0.032) and on intake of specific protein sources, namely, protein intake from meat and meat products (*β* = −0.14, *p* = 0.018), fruit and vegetables (*β* = 0.20, *p* = 0.002), and pulses and nuts and seeds (*β* = 0.16, *p* = 0.016) were identified (Table 4). Whereas participants with stronger HEM had lower intakes of protein from meat and meat products, they showed a higher intake of the two plant-based protein sources. In terms of functional status, a significant inverse relation between SPPB sum score and protein intake from other animal-based sources was revealed.

With regard to possible interaction effects of HEM x functional status, a significant interaction emerged for the intake of plant-based protein (*β* = −0.15, *p* = 0.006) and of the sub-group “fruit and vegetables” (*β* = −0.15, *p* = 0.018). Follow-up analysis revealed that HEM was only positively related to a higher intake of both protein sources among those participants with higher functional status, whereas no significant association of HEM and both protein sources was found among those with lower functional status (see Supplementary Materials, Figures S1 and S2).

## 4. Discussion

To the best of our knowledge, this is the first study that investigates the association of healthy eating motivation (HEM) with aspects of protein intake in community-dwelling older persons varying in functional status. The results showed no relationship between HEM and total protein intake. However, HEM was positively linked to the consumption of plant-based protein, namely, protein from fruit and vegetables and pulses and nuts and seeds, as well as negatively related to protein intake from meat and meat products. Therefore, our results suggest that the strength of health orientation in eating behavior could be a valuable indicator of the consumption of specific protein sources.

The motivation to eat healthily was relatively strong in our participants, independent of functional status [18]. This has already been shown in preliminary studies identifying health concerns as one of the most influencing motives for food choice among older adults [34].

Mean protein intake was 0.9 g/kg BW, which is fairly similar to the results of larger surveys reporting intakes between 0.9–1.0 g/kg BW among community-dwelling, older adults aged ≥65 years and older [12,35,36]. Nevertheless, only 37.6% reached the age-adapted, recommended amount of 1.0 g/kg BW according to the guidelines from the German Nutrition Society [37] and more than 30% had low relative protein intake <0.8 g/kg BW, the amount recommended for younger adults (Table 1). Compared to a Dutch study [38] with 15% community-dwelling older adults having protein intake <0.8 g/kg BW, our group of older adults showed a higher prevalence of low relative protein intake. This is probably because the Dutch study examined a relatively healthy, comparatively younger group (mean age 76 years) underrepresenting older adults with functional impairments and multimorbidity. Surprisingly, in a study also from the UK, including community-dwelling persons aged ≥85 years, revealed a slightly lower prevalence (28%) than our comparatively younger sample [12], which may be due to the targeted inclusion of people with moderate to severe functional impairments in our study. In summary, our results support the need for more targeted public health campaigns addressing sufficient protein intake as part of a health-promoting diet since the respective reference values are often not reached [8,39,40,41].

### 4.1. HEM and Aspects of Protein Intake

Contrary to our first hypothesis, no relation between HEM and total protein intake was identified. Considering the associations between HEM and consumption of different protein sources, an explanation might be that HEM was both positively and negatively associated with the intake from certain protein sources. A protein-rich diet is one of the core components of dietary guidelines [42] and nutritional health promotion strategies addressing older adults. However, knowledge of the importance of protein intake at the individual level might be still insufficient or only available in those who already have a fairly healthy lifestyle. Hence, it might be important to highlight the health-promoting value of high-quality proteins to older adults. Moreover, it could be presumed that available knowledge on the relevance of protein intake for health is not transferred to actual dietary behavior. There are many possible reasons for this: First, studies have shown that although motivation to eat healthy is strong among older adults, ecological and ethical concerns are influencing eating behavior as well [18]. This could limit the consumption of animal foods such as fish or poultry, which are valuable sources of protein but whose consumption is highly controversial from an ecological point of view [43,44]. Second, eating behavior is complex and influenced by many different eating motives [5,28] that may compete at the individual level. In addition to motives for eating, Bloom et al. [45] outlined various factors associated with food choice in older age, such as lifelong food experiences, medical conditions, environmental and psychosocial factors. Third, with advanced age, diminishing physical, financial, and social resources could represent barriers to a health-oriented [46] and protein-rich diet that often requires frequent shopping activities and freshly prepared foods [47]. Another explanation for the missing relationship between HEM and total protein intake might be that HEM was assessed generally with a brief TEMS health motive. One might speculate that more specific aspects of healthy eating motivation, such as a focus on nutrients or even explicitly protein, could be related to total protein intake.

Although HEM was not associated with the intake of animal protein in general, in line with our hypothesis, we found that stronger HEM was significantly related to lower consumption of protein from meat and meat products. An association of health considerations and meat consumption was already shown by Tobler et al. [48], who investigated the willingness to adopt ecological food consumption behavior. The conviction that less meat intake is health-promoting was identified as a motivator for reduced meat intake. Nevertheless, it was also shown that the amount of consumed meat is influenced by various factors, such as ethical and environmental concerns [29,48,49]. Correlations within our protein sources data revealed that lower consumption of protein from meat and meat products was neither linked to intake of protein from the other three animal-based protein sources nor to the consumed amount of plant-based protein sources. These findings contradict a shift from lower meat consumption to a higher intake of food-items from other animal or plant protein sources, as recommended by official dietary guidelines for the general population such as those of the German Nutrition Society [50] and the World Cancer Research Fund International [51].

The significant higher consumption of plant-based protein among those with stronger HEM, reflected in a higher intake of proteins from fruits and vegetables and pulses and nuts and seeds, is in line with previous findings from studies in age-mixed groups [20,52]. It supports the assumption that the perception of healthy eating may be primarily determined by the intake of plant foods [53,54]. Although a frequent intake of fruits and vegetables corresponds to a healthy dietary pattern due to the high content of vitamins and fiber, the protein content of these foods is rather low [17]. As plant-based foods contain, on average, less protein with lower biological quality of the amino acid composition than animal-based foods, larger amounts and a careful choice and combination of protein-rich plant-based foods are needed in case of a plant-based or vegetarian diet to meet protein requirements [55]. In particular, this should be taken into account when a shift from lower consumption of animal-based food to more plant-based food is sought.

In summary, our results suggest that health concerns in eating motivation may be associated with a lower overall intake of foods containing high-quality animal protein sources and a higher intake of foods containing other health-promoting nutrients but little protein. However, it must be considered that the effect sizes of our findings were only small, indicating that protein intake is also determined by other factors. With regard to the clinical importance of our results, future studies with a longitudinal design are needed to investigate the relationship between intake of protein sources associated with HEM and parameters such as functional status or muscle mass in older adults.

### 4.2. Role of Functional Status

Surprisingly, a significant inverse association of functional status and relative protein intake was observed. Participants with better functional status had slightly lower relative protein intake compared to participants with worse functional status. Since the effect of functional status on relative protein intake is very weak, the clinical significance of this finding is questionable. However, we also identified a significantly higher intake of protein from the category “other animal-based protein sources” in functionally impaired participants, a category which mainly contains food items like cakes and desserts as well as ready-meals. These participants might be comparatively more restricted in shopping and cooking activities, which may have led to a higher consumption of processed foods instead of fresh foods containing less protein [56].

Compared to other studies showing evidence for an association between lower protein intake and worse functional status cross-sectionally [10,15], our participants could be more robust, despite the presence of functional limitations. As participation in both presented studies suggested a certain interest in health-related issues, older adults with rather healthy lifestyles and comparatively good resources to deal with losses in physical function are likely to be over-represented.

Considering functional status as potential moderator of the relationship between HEM and intake of protein sources, we observed HEM only being significantly associated with intake of plant-based protein and protein from fruit and vegetables among those with higher physical function. This could lead to the conclusion that if functional performance is poor, the motive of healthy eating can no longer be translated into respective behavior, for example, due to difficulties in obtaining and preparing fresh food [56,57]. Remarkably, however, functionally limited participants with low health orientation in eating have a slightly higher intake of plant protein in our study than more robust participants with the same low HEM. It is possible that these people with functional limitations may receive support in the provision of food, for example, from relatives or friends, which is why their own eating motivation is less reflected in their eating behavior. Animal-based protein sources are regarded as highly relevant for muscle functioning [7,17,58,59]; nevertheless, it is to date still discussed to what extent distinct protein sources affect different domains of physical function. For example, Coelho et al. [60] and Gazzani et al. [61] both identified a positive association of walking speed and plant-based protein ingestion. Future studies are needed to investigate the relationship between different aspects of protein intake and distinct functional performance components.

### 4.3. Practical Implications

First, the considerable proportion of participants with a low protein intake underlines the importance of assessing and promoting protein intake in this population. According to our study results, potential insufficient protein intake should be considered as a limiting factor of a health-promoting diet in old age regardless of the strength of health motivation in eating and functional status. The collection and evaluation of comprehensive food records in individual health care (e.g., in the GP’s practice) and in population-related health campaigns may be difficult to implement for reasons of time and personnel [62,63]. However, the application of fast and economically efficient methods such as the “protein screener” developed by Wijnhoven et al. [64] could be a valuable strategy to identify low protein intakes. In addition, health campaigns could focus on the development and dissemination of methods for quick self-evaluation of one’s protein intake. In case of insufficient protein intake, recommendations to enhance protein consumption should be formulated as concretely as possible. For example, recommendations focusing on integrating foods rich in protein in daily meals might be easier to implement for older people compared to recommendations on the nutrient level.

Second, the missing association between the health motive and overall protein intake underlines the need for an understanding of eating as complex and individual behavior, potentially moderated by various physiological, psychological, and social factors [65]. Consequently, health promotion, and especially nutrition education in older age, should go beyond the focus on health concerns in food choice and take into account individual motives and needs [66].

Third, implications for the development of concrete strategies to promote a protein-rich diet can be derived from our findings on the relationship between HEM and the intake of different protein sources. In older adults, a stronger HEM could be seen as an indicator of a health-conscious diet characterized by a higher intake of plant-based food. Nevertheless, health campaigns should communicate that a healthy diet in terms of protein is easier to achieve by a combination of distinct high-quality plant-based and animal-based protein sources [17]. Due to the environmental and ethical consequences of the production and consumption of animal foods, more rigorous sustainability policies in the meat and dairy industry are needed [67]. In addition, more sustainable options should be offered to meet protein requirements by including vegetarian products and meat analogs [68].

### 4.4. Strength and Limitations

The strength of the present study is the assessment of dietary intake by using prospective 7-day food records. This method allows considering variations in dietary intake, for example, possible changes in food intake during week- and weekend days. Notwithstanding this, self-assessed dietary intake is prone to reporting bias; for instance, energy-dense or sugar-rich foods are often underreported [69]. In order to minimize this risk of underreporting, participants were instructed face-to-face by trained study personal and every protocol was checked for completeness and plausibility. Moreover, we were able to recruit a group of older people that reflects the diversity of functional status among mobile community-living older adults without need of care.

Despite these strengths, we acknowledge several limitations. Firstly, due to the cross-sectional and non-interventional design of this study, no causal relation between HEM and variables of protein intake can be derived. Notwithstanding this, previous studies and models of food choice support the assumption that motivation is an antecedent of eating behavior [70,71]. Secondly, although the consumed protein-containing foods were systematically assigned to the various protein sources in terms of quantity, we were not able to consider differences in the nutritional quality of individual food items within a protein source category. For instance, within the category “meat and meat products”, we did not distinguish between potentially health-harmful foods such as processed or red meat and more health-promoting food items such as poultry. Finally, we could not account for factors such as appetite, oral health, chemosensory losses, social support, and meal provision that might have affected protein intake in our group of older adults [12,65,72].

## 5. Conclusions

While a strong, healthy eating motivation (HEM) was not associated with an overall protein-rich diet in community-dwelling older adults varying in functional status, the results suggest that HEM could serve as an indicator of a pattern of consuming certain protein sources and as an aspect to be incorporated in individually tailored nutrition intervention. Among older adults with strong HEM, it should be communicated that a healthy diet is not only characterized by a high intake of plant-based foods but also requires sufficient consumption of distinct high-quality protein sources. Among those with low levels of HEM, it may be more appropriate to promote a protein-rich diet by using perceptions of tastiness, affordability, and convenience. However, the finding that low protein intake is prevalent in almost one-third of our sample underlines that nutritional health promotion in older age should initially focus on sufficient protein intake, regardless of self-reported healthy eating motivation. Future studies are needed to investigate the association of HEM and aspects of protein intake longitudinally and to gain a deeper insight into older adults’ interpretations of a healthy, protein-rich diet.

## Figures and Tables

**Table 1:** Participants’ characteristics, HEM, and dietary intake for the total sample and separated by gender.

	Total (*N* = 250)		Female (*n* = 144)		Male (*n* = 106)	
	n/mean	%/SD	n/mean	%/SD	n/mean	%/SD
**Characteristic**						
Age [years]	79.3	±4.2	80.1	±4.6	78.3	±3.5
Living alone *	158	63.2%	115	79.9%	43	40.6%
Weight [kg] *	74.3	±16.3	67.4	±15.0	83.5	±13.0
BMI [kg/m^2^]	27.7	±5.1	27.3	±5.6	28.2	±4.3
SPPB [score, 0–12 p.]	9.6	±2.5	9.1	±2.6	10.2	±2.4
HEM [score, 1–7p.]	4.9	±1.5	5.2	±1.5	4.5	±1.4
**Daily dietary intake**						
Energy [kcal] *	1806.1	±418	1652.7	±338.2	2014.5	±427.1
Carbohydrates [g] *	188.9	±51.2	173.8	±41.0	209.5	±56.6
Fat [g] *	77.6	±20.7	73.0	±18.0	83.9	±22.5
Protein [g] *	67.7	±17.7	62.4	±14.3	74.8	±19.3
Protein [g/kg BW]	0.94	±0.28	0.96	±0.27	0.92	±0.28
Protein intake ≥0.8g/kg/BW	174	69.6%	109	75.5%	65	61.3%
Protein intake ≥1.0g/kg/BW	94	37.6%	55	38.2%	39	36.8%
Animal-based protein [g] *	42.1	±15.2	37.7	±12.1	48.0	±16.8
Meat and meat products [g] *	17.9	±11.5	14.8	±8.9	22.2	±13.2
*Dairy products [g]*	13.3	±8.4	13.2	±8.3	13.4	±8.5
*Fish and seafood [g]*	4.9	±4.8	4.3	±4.2	5.5	±5.4
*Other animal-based p. s. [g]*	6.0	±3.9	5.4	±3.2	6.9	±4.6
Plant-based protein [g]	25.6	±8.2	24.7	±7.6	26.8	±8.7
*Starchy foods [g]*	13.6	±5.4	13.1	±5.4	14.1	±5.4
*Fruits and vegetables [g]*	4.8	±3.0	4.9	±2.8	4.7	±3.3
*Pulses and nuts and seeds [g]*	2.2	±3.8	2.4	±4.2	2.0	±3.1
Other plant-based p.s. [g] *	5.0	±3.2	4.3	±2.5	6.0	±3.8

Significant differences refer to gender differences * *p* < 0.002 (Bonferroni-corrected), chi-square-test or *t*-test; BMI, body mass index; HEM, healthy eating motivation; MMSE, Mini Mental Status examination; SPPB, short physical performance battery; p.s., protein sources.

**Table 2:** Pearson correlations of data on protein intake.

		1	2	3	4	5	6	7	8	9	10	11	12	13
Energy	1	1	0.76 *	0.53 *	0.57 *	0.36 *	0.31 *	0.26 *	0.20 *	0.58 *	0.47 *	0.14 *	0.08	0.45 *
Protein [g]	2		1	0.70 *	0.89 *	0.60 *	0.51 *	0.35 *	0.16 *	0.52 *	0.45 *	0.20 *	0.15 *	0.19 *
Protein [g/kg BW]	3			1	0.56 *	0.27 *	0.48 *	0.25 *	0.05	0.47 *	0.39 *	0.20 *	0.23 *	0.09
Animal-based protein [g]	4				1	0.72 *	0.49 *	0.36 *	0.26 *	0.07	0.15 *	−0.02	−0.09	0.05
*Meat and meat products [g]*	5					1	−0.10	0.02	0.03	−0.04	0.08	−0.11	−0.12	0.00
*Dairy products [g]*	6						1	0.06	−0.02	0.10 *	0.16 *	0.18 *	0.04	0.02
*Fish and seafood [g]*	7							1	−0.01	0.08	0.08	0.05	0.04	−0.03
*Other animal-based p.s. [g]*	8								1	−0.14 *	−0.11	−0.19 *	−0.15 *	0.19 *
Plant-based protein [g]	9									1	0.71 *	0.47 *	0.50 *	0.32 *
*Starchy foods [g]*	10										1	0.08	−0.02	0.06
*Fruits and vegetables [g]*	11											1	0.25 *	−0.17 *
*Pulses and nuts and seeds [g]*	12												1	−0.11
*Other plant-based p. s. [g]*	13													1

Pearson correlations are significant at * *p* < 0.05; p.s., protein sources.

**Table 3:** Results of the final step of the hierarchical multiple regression analysis (Model 4) testing the association of HEM and total protein intake (*N* = 250).

	B	SE	95% CI		β	*p*	Model Fit
**Protein [g]**							
*Constant*	28.57	18.50	−7.87	65.01		<0.001	*R*^2^ = 0.596, F(6,243) = 59.86, *p* = < 0.001
*HEM*	−0.18	0.51	−1.18	0.82	−0.02	0.723	
*SPPB*	−0.22	0.33	−0.87	0.43	−0.03	0.508	
*Gender*	−0.04	1.67	−3.34	3.25	0.00	0.980	
*Age*	−0.33	0.19	−0.70	0.04	−0.08	0.076	
*BMI*	0.36	0.16	0.06	0.67	0.11	0.021	
*Energy (kcal)*	0.03	0.00	0.03	0.04	0.76	<0.001	
**Protein [g/kg BW]**							
*Constant*	1.40	0.30	0.81	1.98		<0.001	*R*^2^ = 0.580; F(6,243) = 55.85, *p* = < 0.001
*HEM*	0.01	0.01	−0.01	0.02	0.04	0.370	
*SPPB*	−0.01	0.01	−0.02	0.00	−0.11	0.023	
*Gender*	−0.16	0.03	−0.21	−0.10	−0.28	<0.001	
*Age*	0.00	0.00	−0.01	0.00	−0.04	0.391	
*BMI*	−0.03	0.00	−0.03	−0.02	−0.46	<0.001	
*Energy (kcal)*	0.00	0.00	0.00	0.00	0.62	<0.001	

B, unstandardized beta; SE, standard error; CI, confidence interval; β, standardized beta; HEM, healthy eating motivation; SPPB, short physical performance battery; BMI, body mass index.

**Table 4:** Results of the final step of the hierarchical multiple regression analysis testing the association of HEM and protein intake by animal and plant sources (*N* = 250).

	B	SE	95% CI		β	*p*	Model Fit
**Animal-based protein (g)**							
*Constant*	19.98	19.65	−18.73	58.68		0.310	*R*^2^ = 0.381, F(6,243) = 24.98, *p* = < 0.001
*HEM*	−0.69	0.54	−1.75	0.37	-0.07	0.201	
*SPPB*	−0.17	0.35	−0.86	0.53	-0.03	0.640	
*Gender*	1.86	1.78	−1.64	5.36	0.06	0.297	
*Age*	−0.31	0.20	−0.70	0.08	-0.09	0.122	
*BMI*	0.45	0.17	0.13	0.78	0.15	0.007	
*Energy (kcal)*	0.02	0.00	0.02	0.02	0.55	<0.001	
**Meat and meat products (g)**							
*Constant*	−18.56	16.53	−51.11	14.00		0.263	*R*^2^ = 0.241, F(6,243) = 12.87, *p* = < 0.001
*HEM*	−1.08	0.45	−1.97	−0.19	−0.14	0.018	
*SPPB*	0.09	0.30	−0.50	0.67	0.02	0.770	
*Gender*	3.05	1.50	0.10	5.99	0.13	0.043	
*Age*	0.07	0.17	−0.26	0.40	0.03	0.671	
*BMI*	0.55	0.14	0.27	0.82	0.24	< 0.001	
*Energy* *(kcal)*	0.01	0.00	0.01	0.01	0.32	< 0.001	
**Dairy products (g)**							
*Constant*	23.68	12.78	−1.50	48.86		0.065	*R*^2^ = 0.139, F(6,243) = 6.52, *p* = < 0.001
*HEM*	0.42	0.35	−0.27	1.11	0.07	0.229	
*SPPB*	0.12	0.23	−0.34	0.57	0.04	0.612	
*Gender*	−2.36	1.16	−4.64	−0.08	−0.14	0.042	
*Age*	−0.26	0.13	−0.51	0.00	−0.13	0.048	
*BMI*	−0.07	0.11	−0.28	0.14	−0.04	0.512	
*Energy (kcal)*	0.01	0.00	0.00	0.01	0.33	< 0.001	
**Fish and seafood (g)**							
*Constant*	5.97	7.62	−9.05	20.98		0.434	*R*^2^ = 0.073, F(6,243) = 3.17, *p* = 0.005
*HEM*	−0.19	0.21	−0.60	0.22	−0.06	0.370	
*SPPB*	0.01	0.14	−0.26	0.28	0.01	0.926	
*Gender*	−0.03	0.69	−1.39	1.33	0.00	0.966	
*Age*	−0.06	0.08	−0.21	0.09	−0.05	0.456	
*BMI*	−0.03	0.06	−0.15	0.10	−0.03	0.665	
*Energy (kcal)*	0.00	0.00	0.00	0.00	0.24	0.001	
**Other animal-based p.s. (g)**							
*Constant*	8.88	6.06	−3.06	20.83		0.144	*R*^2^ = 0.106, F(6,243) = 4.81, *p* = < 0.001
*HEM*	0.15	0.17	−0.18	0.48	0.06	0.362	
*SPPB*	−0.38	0.11	−0.60	−0.17	−0.25	0.001	
*Gender*	1.20	0.55	0.12	2.28	0.15	0.030	
*Age*	−0.07	0.06	−0.19	0.05	−0.07	0.284	
*BMI*	0.00	0.05	−0.10	0.11	0.01	0.926	
*Energy (kcal)*	0.00	0.00	0.00	0.00	0.20	0.004	
**Plant-based protein (g)**							
*Constant*	4.80	10.72	−16.32	25.91		0.655	*R*^2^ = 0.381, F(7,242) = 21.27, *p* = < 0.001
*HEM*	0.64	0.29	0.05	1.22	0.11	0.032	
*SPPB*	−0.04	0.19	−0.41	0.34	−0.01	0.851	
HEM*SPPB	1.17	0.42	0.34	1.99	0.15	0.006	
*Gender*	−2.21	0.97	−4.11	−0.31	−0.13	0.023	
*Age*	0.01	0.11	−0.20	0.22	0.00	0.931	
*BMI*	−0.08	0.09	−0.26	0.10	−0.05	0.377	
*Energy (kcal)*	0.01	0.00	0.01	0.01	0.64	<0.001	
**Starchy foods (g)**							
*Constant*	7.54	7.79	−7.80	22.87		0.334	*R*^2^ = 0.244, F(6,243) = 13.08, *p* = < 0.001
*HEM*	−0.32	0.21	−0.74	0.10	−0.09	0.135	
*SPPB*	−0.05	0.14	−0.32	0.23	−0.02	0.728	
*Gender*	−1.57	0.70	−2.96	-0.18	−0.14	0.027	
*Age*	−0.01	0.08	−0.16	0.15	−0.01	0.934	
*BMI*	−0.06	0.07	−0.19	0.07	−0.05	0.384	
*Energy (kcal)*	0.01	0.00	0.01	0.01	0.53	<0.001	
**Fruits and vegetables (g)**							
*Constant*	2.38	4.81	−7.11	11.86		0.622	*R*^2^ = 0.091, F(7,242) = 3.45, *p* = 0.002
*HEM*	0.41	0.13	0.15	0.67	0.20	0.002	
*SPPB*	0.10	0.09	−0.07	0.26	0.08	0.265	
HEM*SPPB	0.45	0.19	0.08	0.82	0.15	0.018	
*Gender*	−0.70	0.43	−1.56	0.15	−0.11	0.107	
*Age*	−0.03	0.05	−0.12	0.07	−0.04	0.580	
*BMI*	0.01	0.04	−0.07	0.09	0.02	0.722	
*Energy (kcal)*	0.00	0.00	0.00	0.00	0.17	0.017	
**Pulses and nuts and seeds (g)**							
*Constant*	4.29	6.10	−7.73	16.31		0.483	*R*^2^ = 0.048, F(6,243) =2.06, *p* = 0.059
*HEM*	0.41	0.17	0.08	0.74	0.16	0.016	
*SPPB*	−0.15	0.11	−0.37	0.06	−0.10	0.161	
*Gender*	−0.35	0.55	−1.44	0.74	−0.05	0.529	
*Age*	−0.03	0.06	−0.15	0.09	−0.03	0.613	
*BMI*	−0.06	0.05	−0.16	0.04	−0.08	0.259	
*Energy (kcal)*	0.00	0.00	0.00	0.00	0.12	0.095	
**Other plant-based p.s. (g)**							
*Constant*	−6.79	4.69	−16.03	2.45		0.149	*R*^2^ = 0.213, F(6,243) = 10.99, *p* = < 0.001
*HEM*	0.06	0.13	−0.19	0.32	0.03	0.634	
*SPPB*	0.06	0.08	−0.11	0.23	0.05	0.479	
*Gender*	0.61	0.42	−0.23	1.44	0.09	0.153	
*Age*	0.05	0.05	−0.04	0.14	0.07	0.288	
*BMI*	0.01	0.04	−0.06	0.09	0.02	0.723	
*Energy (kcal)*	0.00	0.00	0.00	0.00	0.41	<0.001	

B, unstandardized beta; SE, standard error; CI, confidence interval; β, standardized beta; HEM, healthy eating motivation; SPPB, short physical performance battery; BMI, body mass index; p.s., protein sources. Interaction term “HEM*SPPB” was removed from models when not significant.

## Supplementary Materials

The following supplementary materials are available online at https://www.mdpi.com/2072-6643/12/3/662/s1, Table S1: Pearson correlations of independent variables; Table S2: Sample characteristics for the complete cases sample and the dropout sample; Table S3: Results of the single steps of the hierarchical multiple regression analysis testing the association of HEM and variables representing protein intake; Figure S1: Plant protein intake as a function of Healthy Eating Motivation (HEM) and SPPB level; Figure S2: Intake of protein from fruits & vegetables as a function of Healthy Eating Motivation (HEM) and SPPB level.
