# Supplementary material for: The Relationship Between Healthy Eating Motivation and Protein Intake in Community-Dwelling Older Adults With Varying Functional Status
Source: Nutrients. 2020 Feb 28;12(3):662. doi: 10.3390/nu12030662 (PMC7146591; doi:10.3390/nu12030662)
Supplement: Supplementary file 1 [file nutrients-12-00662-s001.pdf]

nutrients

MDPI

Supplementary Materials

Table S1. Pearson correlations of independent variables ($N = 250$).

		1	*2*	*3*	*4*	*5*	*6*
1	HEM	1	0.021	−0.208 **	0.077	−0.179 **	−0.058
2	SPPB		1	0.200 **	−0.320 **	−0.301 **	0.325 **
3	Gender			1	−0.206 **	0.091	0.429 **
4	Age				1	−0.113	−0.198 **
5	BMI					1	−0.113
6	Energy intake [kcal]						1

HEM healthy eating motivation; SPPB Short Physical Performance Battery, BMI body mass index.

Table S2. Sample characteristics for the complete cases sample and the dropout sample.

	Complete Cases Sample ($n = 250$)		Dropout Sample ($n = 23$)	
	n/mean	%/SD	n/mean	%/SD
Gender [female]	144	57.6%	10	43.5%
Age [years]	79.3	±4.2	81.0	±5.8
Living alone	158	63.2%	18	78.3%
Weight [kg]	74.3	±16.3	73.2	±14.7
BMI [kg/m^2]	27.7	±5.1	27.5	±5.1
SPPB [score, 0–12 p.] ***	9.6	±2.5	7.8	±2.6

* $p < 0.05$, ** $p < 0.01$, ***$p < 0.001$, chi-square-test or *t*-test; BMI *body mass index*; SPPB *Short Physical Performance Battery.*

Table S3. Results of the single steps of the hierarchical multiple regression analysis testing the association of HEM and variables representing protein intake (*N* = 250)

Protein [g]		B	SE	95 %CI		β	*p*	R^2	F	p^1
Model 1	*Constant*	72.65	3.89	64.99	80.30		<.001	.007	1.79	.183
	HEM	−1.02	0.76	-2.51	0.48	-0.09	.183			
Model 2	*Constant*	58.83	5.55	47.90	69.77		<.001		6.77	.001
	HEM	-1.07	0.74	-2.54	0.40	-0.09	.152	.052		
	SPPB	1.47	0.43	0.62	2.32	0.21	.001			
Model 3	*Constant*	58.55	5.60	47.52	69.58		<.001		4.56	.004
	HEM	-1.01	0.76	-2.50	0.49	-0.08	.185	.053		
	SPPB	1.47	0.43	0.62	2.32	0.21	.001			
	*HEM*SPPB*	0.49	1.10	-1.67	2.64	0.03	.658			
Model 4	*Constant*	25.97	18.69	-10.85	62.79		.166		51.44	<.001
	HEM	-0.10	0.51	-1.11	0.91	-0.01	.844			
	SPPB	-0.21	0.33	-0.86	0.45	-0.03	.531			
	*HEM*SPPB*	0.72	0.73	-0.73	2.16	0.04	.329	.598		
	Gender	-0.24	1.69	-3.56	3.08	-0.01	.888			
	Age	-0.31	0.19	-0.68	0.06	-0.07	.102			
	BMI	0.37	0.16	0.06	0.68	0.11	.018			
	Energy (kcal)	0.03	0.00	0.03	0.04	0.77	<.001			
Model 4 *	*Constant*	28.57	18.50	-7.87	65.01		<.001		59.86	<.001
	HEM	-0.18	0.51	-1.18	0.82	-0.02	.723			
	SPPB	-0.22	0.33	-0.87	0.43	-0.03	.508			
	Gender	-0.04	1.67	-3.34	3.25	0.00	.980	.596		
	Age	-0.33	0.19	-0.70	0.04	-0.08	.076			
	BMI	0.36	0.16	0.06	0.67	0.11	.021			
	Energy (kcal)	0.03	0.00	0.03	0.04	0.76	<.001			

Protein [g/kg BW]		B	SE	95% CI		β	p	R^2	F	p^1
Model 1	*Constant*	0.81	0.06	0.70	0.93		<.001	.019	4.81	.029
	HEM	0.03	0.01	0.00	0.05	0.14	.029			

Model 2	*Constant*	0.63	0.09	0.45	0.80		<.001	.053	6.93	.001
	HEM	0.03	0.01	0.00	0.05	0.13	.031			
	SPPB	0.02	0.01	0.01	0.03	0.18	.003			
Model 3	*Constant*	0.62	0.09	0.45	0.80		<.001	.053	4.60	.004
	HEM	0.03	0.01	0.00	0.05	0.14	.033			
	SPPB	0.02	0.01	0.01	0.03	0.18	.003			
	*HEM*SPPB*	0.00	0.02	-0.03	0.04	0.01	.918			
Model 4	*Constant*	1.35	0.30	0.76	1.94		<.001	.582	48.09	<.001
	HEM	0.01	0.01	-0.01	0.02	0.05	.290			
	SPPB	-0.01	0.01	-0.02	0.00	-0.11	.025			
	*HEM*SPPB*	0.01	0.01	-0.01	0.04	0.05	.269			
	Gender	-0.16	0.03	-0.21	-0.11	-0.29	<.001			
	Age	0.00	0.00	-0.01	0.00	-0.03	.477			
	BMI	-0.03	0.00	-0.03	-0.02	-0.46	<.001			
	Energy (kcal)	0.00	0.00	0.00	0.00	0.63	<.001			
Model 4 *	*Constant*	1.40	0.30	0.81	1.98		<.001	.580	55.85	<.001
	HEM	0.01	0.01	-0.01	0.02	0.04	.370			
	SPPB	-0.01	0.01	-0.02	0.00	-0.11	.023			
	Gender	-0.16	0.03	-0.21	-0.10	-0.28	<.001			
	Age	0.00	0.00	-0.01	0.00	-0.04	.391			
	BMI	-0.03	0.00	-0.03	-0.02	-0.46	<.001			
	Energy (kcal)	0.00	0.00	0.00	0.00	0.62	<.001			
Animal-based protein [g]		B	SE	95% CI		β	p	R^2	F	p[1]
Model 1	*Constant*	49.42	3.31	42.90	55.94		<.001	.021	5.41	.021
	HEM	-1.51	0.65	-2.78	-0.23	-0.15	.021			
Model 2	*Constant*	41.16	4.79	31.74	50.59		<.001	.043	5.56	.004
	HEM	-1.54	0.64	-2.80	-0.27	-0.15	.017			
	SPPB	0.88	0.37	0.15	1.61	0.15	.019			
Model 3	*Constant*	41.41	4.83	31.91	50.91		<.001	.044	3.76	.011
	HEM	-1.59	0.65	-2.88	-0.30	-0.15	.016			

	SPPB	0.88	0.37	0.15	1.61	0.15	.019			
	*HEM*SPPB*	-0.42	0.94	-2.28	1.44	-0.03	.658			
Model 4	*Constant*	21.61	19.88	-17.55	60.76		.278		21.40	<.001
	HEM	-0.74	0.55	-1.82	0.34	-0.07	.177			
	SPPB	-0.17	0.35	-0.87	0.52	-0.03	.626			
	*HEM*SPPB*	-0.45	0.78	-1.98	1.09	-0.03	.566	.382		
	Gender	1.98	1.79	-1.55	5.51	0.06	.270			
	Age	-0.32	0.20	-0.71	0.07	-0.09	.108			
	BMI	0.45	0.17	0.12	0.78	0.15	.008			
	Energy (kcal)	0.02	0.00	0.02	0.02	0.55	<.001			
Model 4 *	*Constant*	19.98	19.65	-18.73	58.68		.310		24.98	<.001
	HEM	-0.69	0.54	-1.75	0.37	-0.07	.201			
	SPPB	-0.17	0.35	-0.86	0.53	-0.03	.640			
	Gender	1.86	1.78	-1.64	5.36	0.06	.297	.381		
	Age	-0.31	0.20	-0.70	0.08	-0.09	.122			
	BMI	0.45	0.17	0.13	0.78	0.15	.007			
	Energy (kcal)	0.02	0.00	0.02	0.02	0.55	<.001			

Plant-based protein [g]		B	SE	95% CI		β	p	R^2	F	p[1]
Model 1	*Constant*	23.23	1.79	19.69	26.76		<.001	.008	1.91	.168
	HEM	0.49	0.35	-0.21	1.18	0.09	.168			
Model 2	*Constant*	17.63	2.58	12.55	22.71		<.001		5.43	.005
	HEM	0.46	0.35	-0.22	1.15	0.08	.181	.042		
	SPPB	0.60	0.20	0.20	0.99	0.19	.003			
Model 3	*Constant*	17.10	2.58	12.01	22.19		<.001		4.73	.003
	HEM	0.58	0.35	-0.11	1.27	0.10	.099	.055		
	SPPB	0.59	0.20	0.20	0.98	0.18	.003			
	*HEM*SPPB*	0.91	0.51	-0.09	1.91	0.11	.073			
Model 4	*Constant*	4.80	10.72	-16.32	25.91		.655		21.27	<.001
	HEM	0.64	0.29	0.05	1.22	0.11	.032	.381		
	SPPB	-0.04	0.19	-0.41	0.34	-0.01	.851			

	*HEM*SPPB*	1.17	0.42	0.34	1.99	0.15	.006			
	Gender	-2.21	0.97	-4.11	-0.31	-0.13	.023			
	Age	0.01	0.11	-0.20	0.22	0.00	.931			
	BMI	-0.08	0.09	-0.26	0.10	-0.05	.377			
	Energy (kcal)	0.01	0.00	0.01	0.01	0.64	<.001			

Meat and meat products [g]		B	SE	95% CI		β	p	R^2	F	p[1]
Model 1	*Constant*	26.54	2.48	21.66	31.42		<.001	.050	13.14	<.001
	HEM	-1.76	0.48	-2.71	-0.80	-0.22	<.001			
Model 2	*Constant*	23.57	3.61	16.46	30.69		<.001		7.22	.001
	HEM	-1.77	0.48	-2.72	-0.81	-0.23	<.001	.055		
	SPPB	0.32	0.28	-0.24	0.87	0.07	.260			
Model 3	*Constant*	23.56	3.64	16.39	30.74		<.001		4.79	.003
	HEM	-1.77	0.49	-2.74	-0.79	-0.23	<.001	.055		
	SPPB	0.32	0.28	-0.24	0.87	0.07	.262			
	*HEM*SPPB*	0.02	0.71	-1.39	1.42	0.00	.981			
Model 4	*Constant*	-18.60	16.73	-51.56	14.36		.267		10.99	<.001
	HEM	-1.08	0.46	-1.98	-0.17	-0.14	.020			
	SPPB	0.09	0.30	-0.50	0.67	0.02	.770			
	*HEM*SPPB*	0.01	0.66	-1.28	1.30	0.00	.987	.241		
	Gender	3.05	1.51	0.07	6.02	0.13	.045			
	Age	0.07	0.17	-0.26	0.40	0.03	.672			
	BMI	0.55	0.14	0.27	0.82	0.24	<.001			
	Energy (kcal)	0.01	0.00	0.01	0.01	0.32	<.001			
Model 4 *	*Constant*	-18.56	16.53	-51.11	14.00		.263		12.87	<.001
	HEM	-1.08	0.45	-1.97	-0.19	-0.14	.018			
	SPPB	0.09	0.30	-0.50	0.67	0.02	.770	.241		
	Gender	3.05	1.50	0.10	5.99	0.13	.043			
	Age	0.07	0.17	-0.26	0.40	0.03	.671			
	BMI	0.55	0.14	0.27	0.82	0.24	<.001			
	Energy (kcal)	0.01	0.00	0.01	0.01	0.32	<.001			

Diary and dairy products [g]		B	SE	95% CI		β	p	R^2	F	p[1]
Model 1	*Constant*	10.99	1.84	7.37	14.61		<.001	.007	1.71	.192
	HEM	0.47	0.36	-0.24	1.18	0.08	.192			
Model 2	*Constant*	5.76	2.65	0.54	10.98		.031	.036	4.56	.011
	HEM	0.45	0.36	-0.25	1.15	0.08	.206			
	SPPB	0.56	0.21	0.15	0.96	0.17	.007			
Model 3	*Constant*	5.81	2.67	0.55	11.07		.031	.036	3.03	.030
	HEM	0.44	0.36	-0.27	1.15	0.08	.226			
	SPPB	0.56	0.21	0.15	0.96	0.17	.007			
	*HEM*SPPB*	-0.09	0.52	-1.12	0.94	-0.01	.870			
Model 4	*Constant*	23.78	12.94	-1.72	49.27		.067	.139	5.57	<.001
	HEM	0.42	0.36	-0.28	1.12	0.07	.239			
	SPPB	0.12	0.23	-0.34	0.57	0.04	.614			
	*HEM*SPPB*	-0.03	0.51	-1.02	0.97	0.00	.960			
	Gender	-2.35	1.17	-4.65	-0.05	-0.14	.045			
	Age	-0.26	0.13	-0.51	0.00	-0.13	.050			
	BMI	-0.07	0.11	-0.28	0.14	-0.04	.512			
	Energy (kcal)	0.01	0.00	0.00	0.01	0.33	<.001			
Model 4 *	*Constant*	23.68	12.78	-1.50	48.86		.065	.139	6.52	<.001
	HEM	0.42	0.35	-0.27	1.11	0.07	.229			
	SPPB	0.12	0.23	-0.34	0.57	0.04	.612			
	Gender	-2.36	1.16	-4.64	-0.08	-0.14	.042			
	Age	-0.26	0.13	-0.51	0.00	-0.13	.048			
	BMI	-0.07	0.11	-0.28	0.14	-0.04	.512			
	Energy (kcal)	0.01	0.00	0.00	0.01	0.33	<.001			

Fish and Seafood [g]		B	SE	95% CI		β	p	R^2	F	p[1]
Model 1	*Constant*	5.90	1.06	3.82	7.98		<.001	.005	1.20	.275
	HEM	-0.23	0.21	-0.63	0.18	-0.07	.275			
Model 2	*Constant*	3.96	1.54	0.93	6.99		.011	.017	2.11	.124
	HEM	-0.23	0.21	-0.64	0.17	-0.07	.258			

	SPPB	0.21	0.12	-0.03	0.44	0.11	.084			
Model 3	*Constant*	4.09	1.55	1.04	7.14		.009		1.58	.196
	HEM	-0.26	0.21	-0.68	0.15	-0.08	.214	.019		
	SPPB	0.21	0.12	-0.03	0.44	0.11	.083			
	*HEM*SPPB*	-0.22	0.30	-0.82	0.38	-0.05	.471			
Model 4	*Constant*	6.78	7.71	-8.40	21.96		.380		2.79	.008
	HEM	-0.21	0.21	-0.63	0.21	-0.07	.317			
	SPPB	0.01	0.14	-0.26	0.28	0.00	.947			
	*HEM*SPPB*	-0.22	0.30	-0.82	0.37	-0.05	.461	.075		
	Gender	0.03	0.70	-1.34	1.40	0.00	.964			
	Age	-0.06	0.08	-0.22	0.09	-0.06	.405			
	BMI	-0.03	0.06	-0.16	0.10	-0.03	.639			
	Energy (kcal)	0.00	0.00	0.00	0.00	0.24	.001			
Model 4 *	*Constant*	5.97	7.62	-9.05	20.98		.434		3.17	.005
	HEM	-0.19	0.21	-0.60	0.22	-0.06	.370			
	SPPB	0.01	0.14	-0.26	0.28	0.01	.926			
	Gender	-0.03	0.69	-1.39	1.33	0.00	.966	.073		
	Age	-0.06	0.08	-0.21	0.09	-0.05	.456			
	BMI	-0.03	0.06	-0.15	0.10	-0.03	.665			
	Energy (kcal)	0.00	0.00	0.00	0.00	0.24	.001			

Other animal-based p. s. [g]		B	SE	95% CI		β	p	R^2	F	p[1]
Model 1	*Constant*	5.98	0.86	4.29	7.67		<.001	.000	0.00	.973
	HEM	0.01	0.17	-0.33	0.34	0.00	.973			
Model 2	*Constant*	7.87	1.25	5.42	10.32		<.001		2.16	.117
	HEM	0.01	0.17	-0.32	0.34	0.00	.937	.017		
	SPPB	-0.20	0.10	-0.39	-0.01	-0.13	.039			
Model 3	*Constant*	7.94	1.26	5.47	10.42		<.001		1.53	.207
	HEM	0.00	0.17	-0.34	0.33	0.00	.983	.018		
	SPPB	-0.20	0.10	-0.39	-0.01	-0.13	.040			
	*HEM*SPPB*	-0.13	0.25	-0.62	0.35	-0.03	.593			

Model 4	*Constant*	9.65	6.13	-2.42	21.72		.117		4.23	<.001
	HEM	0.13	0.17	-0.20	0.46	0.05	.445			
	SPPB	-0.38	0.11	-0.60	-0.17	-0.25	<.001			
	*HEM*SPPB*	-0.21	0.24	-0.68	0.26	-0.05	.382	.109		
	Gender	1.26	0.55	0.17	2.34	0.16	.024			
	Age	-0.07	0.06	-0.19	0.05	-0.08	.241			
	BMI	0.00	0.05	-0.10	0.10	0.00	.961			
	Energy (kcal)	0.00	0.00	0.00	0.00	0.20	.006			
Model 4 *	*Constant*	8.88	6.06	-3.06	20.83		.144		4.81	<.001
	HEM	0.15	0.17	-0.18	0.48	0.06	.362			
	SPPB	-0.38	0.11	-0.60	-0.17	-0.25	.001			
	Gender	1.20	0.55	0.12	2.28	0.15	.030	.106		
	Age	-0.07	0.06	-0.19	0.05	-0.07	.284			
	BMI	0.00	0.05	-0.10	0.11	0.01	.926			
	Energy (kcal)	0.00	0.00	0.00	0.00	0.20	.004			
Starchy foods [g]		B	SE	95% CI		β	p	R^2	F	p[1]
Model 1	*Constant*	14.97	1.20	12.61	17.32		<.001	.006	1.55	.215
	HEM	-0.29	0.23	-0.75	0.17	-0.08	.215			
Model 2	*Constant*	12.22	1.73	8.81	15.63		<.001		3.17	.044
	HEM	-0.30	0.23	-0.76	0.16	-0.08	.195	.025		
	SPPB	0.29	0.13	0.03	0.56	0.14	.030			
Model 3	*Constant*	12.01	1.74	8.58	15.45		<.001		2.46	.064
	HEM	-0.26	0.24	-0.72	0.21	-0.07	.276	.029		
	SPPB	0.29	0.13	0.03	0.56	0.14	.031			
	*HEM*SPPB*	0.35	0.34	-0.33	1.02	0.06	.311			
Model 4	*Constant*	5.66	7.84	-9.77	21.10		.471		11.70	<.001
	HEM	-0.26	0.22	-0.69	0.16	-0.07	.223			
	SPPB	-0.04	0.14	-0.31	0.23	-0.02	.773	.253		
	*HEM*SPPB*	0.52	0.31	-0.09	1.12	0.10	.094			
	Gender	-1.71	0.71	-3.10	-0.32	-0.16	.016			

	Age	0.01	0.08	-0.14	0.17	0.01	.895			
	BMI	-0.05	0.07	-0.18	0.08	-0.05	.431			
	Energy (kcal)	0.01	0.00	0.01	0.01	0.54	<.001			
Model 4 *	*Constant*	7.54	7.79	-7.80	22.87		.334		13.08	<.001
	HEM	-0.32	0.21	-0.74	0.10	-0.09	.135			
	SPPB	-0.05	0.14	-0.32	0.23	-0.02	.728			
	Gender	-1.57	0.70	-2.96	-0.18	-0.14	.027	.244		
	Age	-0.01	0.08	-0.16	0.15	-0.01	.934			
	BMI	-0.06	0.07	-0.19	0.07	-0.05	.384			
	Energy (kcal)	0.01	0.00	0.01	0.01	0.53	<.001			
Fruits & vegetables [g]		B	SE	95% CI		β	p	R^2	F	p[1]
Model 1	*Constant*	3.00	0.66	1.71	4.30		<.001	.032	8.23	.004
	HEM	0.37	0.13	0.12	0.62	0.18	.004			
Model 2	*Constant*	1.66	0.95	-0.21	3.54		.082		6.02	.003
	HEM	0.36	0.13	0.11	0.62	0.18	.005	.046		
	SPPB	0.14	0.07	0.00	0.29	0.12	.055			
Model 3	*Constant*	1.42	0.95	-0.45	3.30		.137		5.72	.001
	HEM	0.42	0.13	0.16	0.67	0.20	.001	.065		
	SPPB	0.14	0.07	0.00	0.28	0.12	.057			
	*HEM*SPPB*	0.41	0.19	0.05	0.78	0.14	.027			
Model 4	*Constant*	2.38	4.81	-7.11	11.86		.622		3.45	.002
	HEM	0.41	0.13	0.15	0.67	0.20	.002			
	SPPB	0.10	0.09	-0.07	0.26	0.08	.265			
	*HEM*SPPB*	0.45	0.19	0.08	0.82	0.15	.018	.091		
	Gender	-0.70	0.43	-1.56	0.15	-0.11	.107			
	Age	-0.03	0.05	-0.12	0.07	-0.04	.580			
	BMI	0.01	0.04	-0.07	0.09	0.02	.722			
	Energy (kcal)	0.00	0.00	0.00	0.00	0.17	.017			
Pulses & nuts & seeds [g]		B	SE	95% CI		β	p	R^2	F	p[1]
	Constant	0.08	0.83	-1.54	1.71		.918		7.31	.007

Model 1	*HEM*	0.44	0.16	0.12	0.76	0.17	.007	.029		
Model 2	*Constant*	0.63	1.21	-1.75	3.00		.605		3.83	.023
	HEM	0.44	0.16	0.12	0.76	0.17	.007	.030		
	SPPB	-0.06	0.09	-0.24	0.13	-0.04	.539			
Model 3	*Constant*	0.54	1.22	-1.85	2.94		.655		2.66	.049
	HEM	0.46	0.16	0.13	0.78	0.18	.006	.031		
	SPPB	-0.06	0.09	-0.24	0.13	-0.04	.533			
	*HEM*SPPB*	0.14	0.24	-0.33	0.61	0.04	.559			
Model 4	*Constant*	3.72	6.17	-8.44	15.87		.548		1.82	.084
	HEM	0.42	0.17	0.09	0.76	0.16	.013			
	SPPB	-0.15	0.11	-0.37	0.06	-0.10	.169			
	*HEM*SPPB*	0.16	0.24	-0.32	0.63	0.04	.515	.050		
	Gender	-0.39	0.56	-1.49	0.71	-0.05	.483			
	Age	-0.03	0.06	-0.15	0.10	-0.03	.676			
	BMI	-0.06	0.05	-0.16	0.05	-0.08	.274			
	Energy (kcal)	0.00	0.00	0.00	0.00	0.13	.086			
Model 4 *	*Constant*	4.29	6.10	-7.73	16.31		.483		2.06	.059
	HEM	0.41	0.17	0.08	0.74	0.16	.016			
	SPPB	-0.15	0.11	-0.37	0.06	-0.10	.161			
	Gender	-0.35	0.55	-1.44	0.74	-0.05	.529	.048		
	Age	-0.03	0.06	-0.15	0.09	-0.03	.613			
	BMI	-0.06	0.05	-0.16	0.04	-0.08	.259			
	Energy (kcal)	0.00	0.00	0.00	0.00	0.12	.095			
Other plant-based p.s. [g]		B	SE	95% CI		β	p	R^2	F	p[1]
Model 1	*Constant*	5.17	0.71	3.78	6.57		<.001	.000	0.04	.837
	HEM	-0.03	0.14	-0.30	0.24	-0.01	.837			
Model 2	*Constant*	3.13	1.02	1.12	5.13		.002		3.82	.023
	HEM	-0.04	0.14	-0.31	0.23	-0.02	.789	.030		
	SPPB	0.22	0.08	0.06	0.37	0.17	.006			
	Constant	3.12	1.03	1.09	5.14		.003		2.53	.057

Model 3	*HEM*	-0.04	0.14	-0.31	0.24	-0.02	.801			
	SPPB	0.22	0.08	0.06	0.37	0.17	.006	.030		
	*HEM*SPPB*	0.01	0.20	-0.39	0.41	0.00	.954			
Model 4	*Constant*	-6.95	4.75	-16.31	2.40		.144		9.39	. <.001
	HEM	0.07	0.13	-0.19	0.32	0.03	.612			
	SPPB	0.06	0.08	-0.11	0.23	0.05	.475			
	*HEM*SPPB*	0.04	0.19	-0.32	0.41	0.01	.810	.214		
	Gender	0.60	0.43	-0.25	1.44	0.09	.165			
	Age	0.05	0.05	-0.04	0.15	0.07	.279			
	BMI	0.01	0.04	-0.06	0.09	0.02	.715			
	Energy (kcal)	0.00	0.00	0.00	0.00	0.41	<.001			
Model 4 *	*Constant*	-6.79	4.69	-16.03	2.45		.149		10.99	<.001
	HEM	0.06	0.13	-0.19	0.32	0.03	.634			
	SPPB	0.06	0.08	-0.11	0.23	0.05	.479	.213		
	Gender	0.61	0.42	-0.23	1.44	0.09	.153			
	Age	0.05	0.05	-0.04	0.14	0.07	.288			
	BMI	0.01	0.04	-0.06	0.09	0.02	.723			
	Energy (kcal)	0.00	0.00	0.00	0.00	0.41	<.001			

B *unstandardized beta*; SE *standard error*; CI *confidence interval*; β *standardized beta*; HEM *healthy eating motivation*; SPPB *Short Physical Performance Battery*; BMI *body mass index*; p.s. *protein sources*. * Interaction term HEM*SPPB was removed from models when not significant. [1] *p*-value for whole model.

Figure S1. Plant protein intake as a function of Healthy Eating Motivation (HEM) and SPPB level.

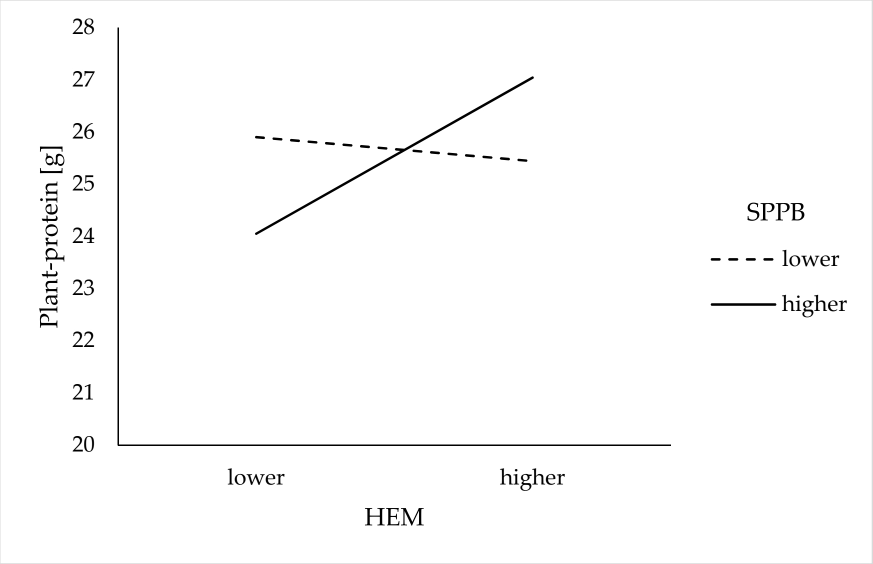

Higher and lower refer to 1 SD below and above the mean; moderator value (SPPB) defining Johnson Neyman significance region = SPPB sum score ≥ 9.4, 42.8% values below, 57.2% values above, $p = 0.05$.

Figure S2. Intake of protein from fruits & vegetables as a function of Healthy Eating Motivation (HEM) and SPPB level.

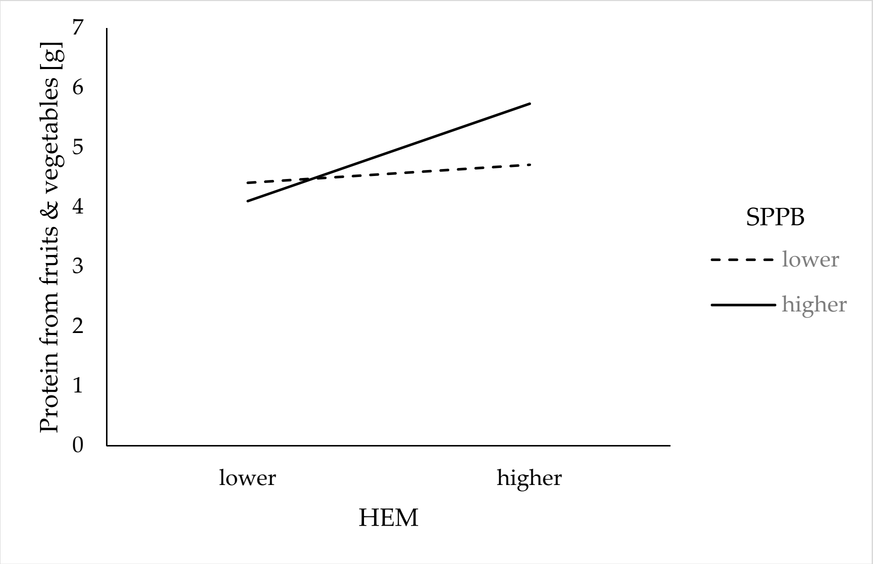

Higher and lower refer to 1 SD below and above the mean; moderator value (SPPB) defining Johnson Neyman significance region = SPPB sum score ≥ 8.4, 35.6% values below, 64.4% values above, $p = 0.05$.
